# Face and content validity of a virtual-reality simulator for myringotomy with tube placement

**DOI:** 10.1186/s40463-015-0094-2

**Published:** 2015-10-20

**Authors:** Caiwen Huang, Horace Cheng, Yves Bureau, Sumit K. Agrawal, Hanif M. Ladak

**Affiliations:** Department of Electrical and Computer Engineering, Western University, London, ON Canada; Department of Otolaryngology – Head and Neck Surgery, Schulich School of Medicine and Dentistry, Western University, London, ON Canada; Biomedical Engineering Graduate Program, Western University, London, ON Canada; Lawson Health Research Institute, London, ON Canada; Department of Medical Biophysics, Western University, London, ON Canada; London Health Sciences Centre, Room B1-333, University Hospital, 339 Windermere Rd., London, N6A 5A5 ON Canada

**Keywords:** Myringotomy, Education, Simulator, Virtual reality, Face validity

## Abstract

**Background:**

Myringotomy with tube insertion can be challenging for junior Otolaryngology residents as it is one of the first microscopic procedures they encounter. The Western myringotomy simulator was developed to allow trainees to practice microscope positioning, myringotomy, and tube placement. This virtual-reality simulator is viewed in stereoscopic 3D, and a haptic device is used to manipulate the digital ear model and surgical tools.

**Objective:**

To assess the face and content validity of the Western myringotomy simulator.

**Methods:**

The myringotomy simulator was integrated with new modules to allow speculum placement, manipulation of an operative microscope, and insertion of the ventilation tube through a deformable tympanic membrane. A questionnaire was developed in consultation with instructing surgeons. Fourteen face validity questions focused on the anatomy of the ear, simulation of the operative microscope, appearance and movement of the surgical instruments, deformation and cutting of the eardrum, and myringotomy tube insertion. Six content validity questions focused on training potential on surgical tasks such as speculum placement, microscope positioning, tool navigation, ear anatomy, myringotomy creation and tube insertion. A total of 12 participants from the Department of Otolaryngology—Head and Neck Surgery were recruited for the study. Prior to completing the questionnaire, participants were oriented to the simulator and given unlimited time to practice until they were comfortable with all of its aspects.

**Results:**

Responses to 12 of the 14 questions on face validity were predominantly positive. One issue of concern was with contact modeling related to tube insertion into the eardrum, and the second was with the movement of the blade and forceps. The former could be resolved by using a higher resolution digital model for the eardrum to improve contact localization. The latter could be resolved by using a higher fidelity haptic device. With regard to content validity, 64 % of the responses were positive, 21 % were neutral, and 15 % were negative.

**Conclusions:**

The Western myringotomy simulator appears to have sufficient face and content validity. Further development with automated metrics and skills transference testing is planned.

## Introduction

Myringotomy with tube insertion is one of the most common procedures in Otolaryngology—Head & Neck Surgery, and is encountered by residents throughout their training. Despite the fact that it is a ubiquitous procedure, the instruction of junior trainees, who often have little experience in microscopic procedures, is often challenging. Montague et al. [1] have analyzed surgical errors through video analysis of actual procedures and note that the 4 most frequently occurring errors in order from most to least occurring include (1) failure to perform a unidirectional myringotomy, (2) making multiple attempts to place the tube, (3) making multiple attempts to complete the myringotomy, and (4) setting the microscope magnification too high. More serious intraoperative complications can also occur including external auditory canal lacerations, medial displacement of tubes into the middle ear, and vascular injuries [2–4]. Although surgical residents can eventually perform standard cases well, they often struggle with narrow canals, retracted tympanic membranes, T-tubes, and procedures performed under local anaesthestic. The goal of simulation is to decrease the learning curve prior to entering the operating, minimize complications in patients, and provide the ability to practice difficult cases.

Several physical models have been described in the literature to provide practice without potential harm to patients [5–9]. Generally, these consist of a tube to mimic the ear canal with a synthetic membrane attached to one end to represent the eardrum. These models do not appear to have gained general acceptance in residency programs, presumably because they are not able to represent anatomical variability easily and the mechanical properties of the materials used do not mimic that of the actual tissues.

Compared with physical models, simulators based on virtual-reality (VR) technologies have the ability to simulate difficult anatomy, model various pathologies, provide automated feedback, and even allow trainees to practice on patient-specific models generated from CT/MRI scans. VR-based simulators have been applied in Otolaryngology, especially for endoscopic sinus surgery [10–14] and for temporal bone drilling [15–18].

In VR simulators, the trainee interacts with realistic 3D digital models of anatomical structures and views them using 3D displays. Simulated tissues can be operated upon using digital representations of actual surgical tools that can be moved in the workspace using devices such as a haptic arm. The sensation of contact force between a digital surgical tool and simulated tissue can be computed and applied to the trainee’s hand via the haptic arm.

The Auditory Biophysics Laboratory at Western University has developed and reported on several aspects of VR-based myringotomy simulation. A blade navigation software system [19, 20] and a system for real-time deformation and cutting of the tympanic membrane [21] were implemented on different software platforms as separate training modules. These versions of the simulator were not integrated and they did not include speculum placement, operating microscope controls for positioning/zooming, or tube insertion through the myringotomy.

As recently reported [22], the Western myringotomy simulator has integrated the previous modules into a common software platform. Moreover, new software modules have been added to allow the user to adjust their surgical view through positioning and tilting of the virtual speculum and operative microscope, and to allow insertion of a ventilation tube into the myringotomy created in a deformable tympanic membrane. The goal is to further expand this simulator in the future to allow trainees to raise tympanomeatal flaps andto eventually perform tympanoplasty/ossiculoplasty on patient-specific anatomy.

In order for training simulators to be accepted into a residency curriculum, a variety of validation studies need to be conducted starting with face validity and culminating in the demonstration that skills acquired in the VR environment transfer to the OR (operating room) environment. Face validity refers to the degree to which a simulation appears like the real situation [23] and content validity measures whether the simulator would be appropriate or useful in training [24, 25]. Although face validity has previously been established for individual software modules [19–21], validation testing has not been performed on the current integrated system, which simulates the entire procedure from microscope positioning to ventilation tube insertion [22].

The objective of this paper is to determine the face and content validity of the new integrated Western myringotomy simulator.

## Methods

### Simulator

**Fig. 1:**
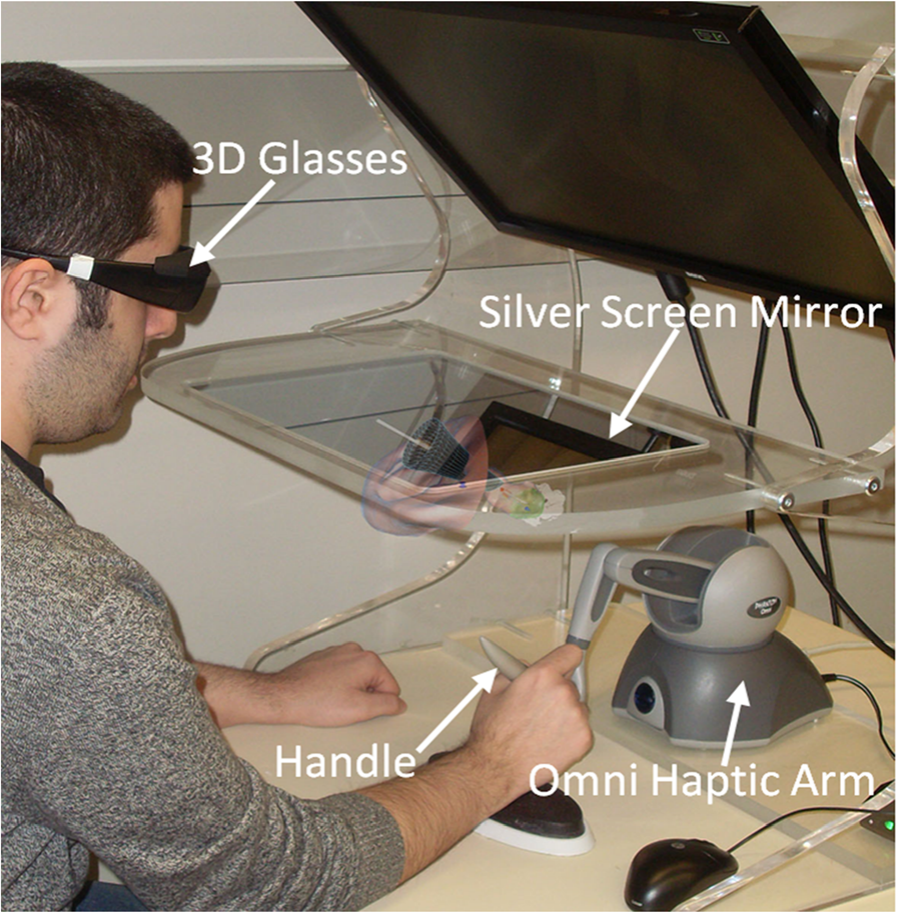
Simulator set up. A user is shown using the Western myringotomy simulator. By moving the handle of the haptic arm, the user controls the movement of a virtual myringotomy blade and forceps. The virtual ear and tools floating under the silver screen mirror are an artistic rendering of what the user would see through the 3D glasses

**Fig. 2:**
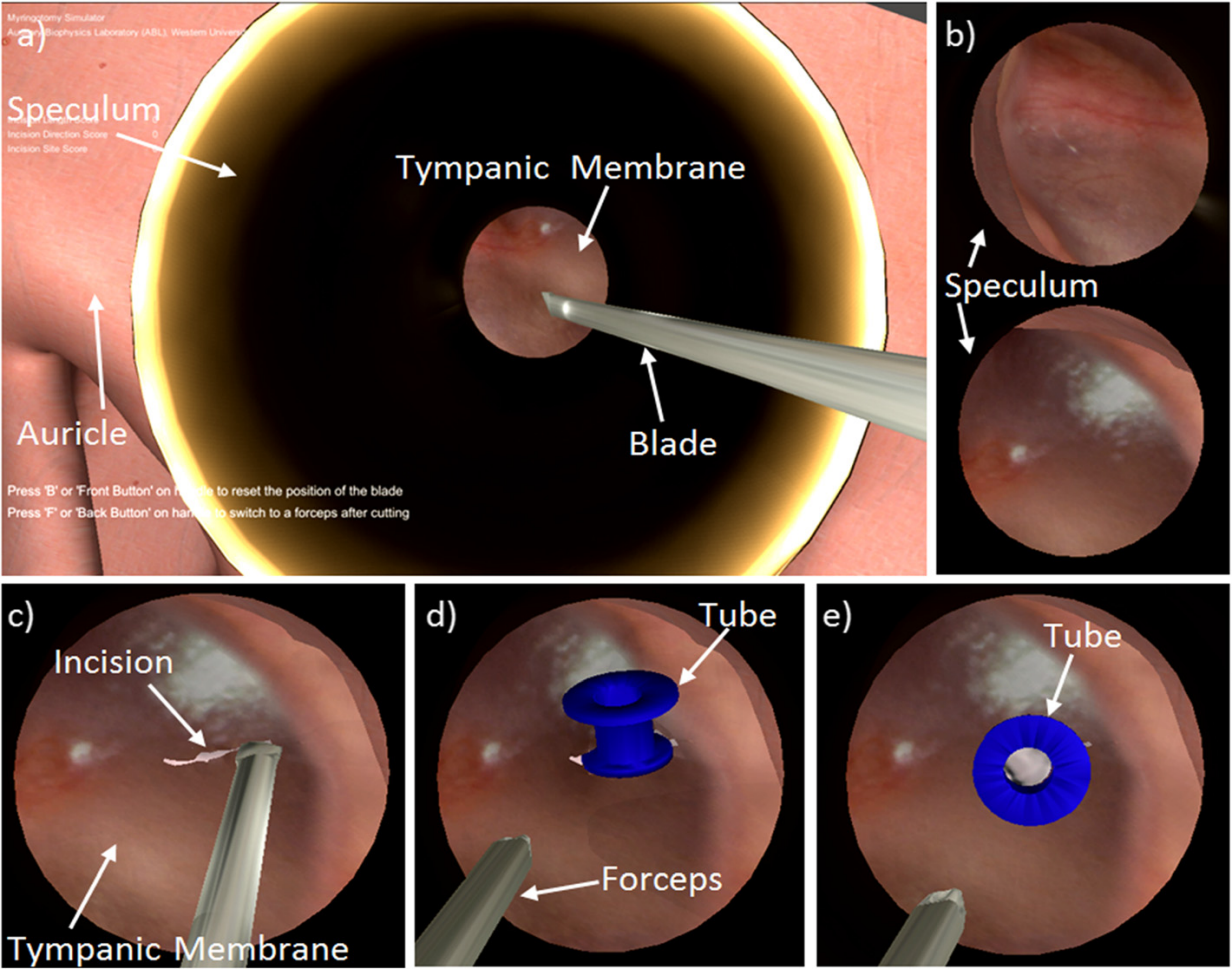
Simulator scene shown in 2D. The actual scene would be viewed by the user in stereoscopic 3D. **a**) View of the speculum and myringotomy blade. **b**) Magnified views of the tympanic membrane through the speculum (represented by the black circle). The view changes depending on the (i) magnification and (ii) position and tilt of the speculum and microscope. **c**) Myringotomy **d**) Tube insertion and splaying of the incision. **e**) Tube in final position with middle ear visible through the lumen of the tube

An overview of the major features of the simulator is given here; in-depth technical details on the system can be found in a previous publication [22]. The simulator consists of 3 major components: the simulation software, a display system, and a haptic arm as shown in Fig. 1. The simulation software was developed in the Auditory Biophysics Laboratory at Western University [19–22]. The simulator runs on a Z420 Hewitt-Packard personal computer, equipped with an Intel(R) Xeon E5-1620 processor (Intel Corp., Sanata Clara, CA) and a NVIDIA Quadro 4000 graphics card (NVIDIA Corp., Santa Clara, CA). The system is capable of real-time rendering of the 3D digital models of the ear, surgical tools, and tympanic membrane as shown in Fig. 2a. The simulator can import various ear canal and tympanic membrane models, however for the purposes of this study, a normal pediatric ear canal and tympanic membrane was used. The system also incorporates multi-point collision detection to monitor for all interactions between the virtual tools and virtual ear and performs real-time deformation and tissue cutting as required. The software displays the models and all interactions on a silver screen mirror that is part of the DevinSense Display 300 system (DevinSense Display Solutions, Sundbyberg, Sweden). When the screen is viewed using active 3D glasses (Nvidia Corp., Santa Clara, CA) provided with the DevinSense system, the 3D digital scene consisting of the virtual ear and tools appears to exist in the space below the silver screen mirror. The display in this region is correctly co-located with the haptic arm (Omni haptic arm, Geomagic, Inc., Morrisville, NC) so movements of the haptic arm appear to occur in the same space as the 3D scene. Using the haptic arm, the user can move the virtual surgical tools. Currently, a single haptic arm is used to control the various instruments, however a second haptic arm could be added to simultaneously manipulate multiple instruments (e.g. speculum and myringotomy blade).

The haptic arm can be used to position and rotate the virtual speculum, position and tilt the microscope, and adjust magnification to obtain different views of the operative site as shown in Fig. 2b. The user can then create a myringotomy as shown in Fig. 2c using a virtual myringotomy blade; the position and orientation of the blade are controlled by moving the handle of the haptic arm. A tube may be inserted using virtual forceps, which is also controlled by the user using the haptic device [Fig. 2d]. The opening and closing of the forceps can be toggled using a button on the haptic arm. During tube insertion, the eardrum deforms and the incision splays as the tube enters the myringotomy. The tube may also be repositioned with various instruments until it is in its final position [Fig. 2e].

### Participants

Research ethics board approval was obtained from Western University (#105239) and participants were contacted via telephone or electronic mail. All participants were recruited from the Department of Otolaryngology - Head & Neck Surgery, Western University. A total of 12 subjects agreed to participate, which included seven junior Otolaryngology residents (postgraduate years 1 to 3) and five senior Otolaryngologists who routinely performed ventilation tube insertions in their practice. These groups were chosen to reflect the target group of the simulator (junior residents) as well as experts in the field (Otolaryngologists). The participants did not have any previous exposure to myringotomy simulation.

### Protocol

All participants were initially given an orientation session which consisted of: 1) an information sheet outlining the software features of the simulator, 2) a demonstration video of how to perform a myringotomy and tube insertion using the simulator controls, and 3) a live demonstration of the simulator and haptic arm. The same graduate student and surgical resident performed the orientation session for each participant, and a standardized script was used to ensure consistency. The participants were specifically asked to perform the tasks listed in Table 1 so that they could comment on all the various aspects of the simulator. Finally, the participants were given an unlimited period of time to use the simulator until they felt comfortable completing the face and content validity questionnaires.

**Table 1:** Tasks involved in the face validity study

Tasks	Description
Speculum adjustment	Rotate and tilt the speculum to obtain view of tympanic membrane
Microscope manipulation	Translate and rotate the microscope to obtain a proper view
Blade navigation	Navigate surgical blade through the external auditory canal
Myringotomy	Make an incision in the tympanic membrane
Ventilation tube insertion	Insert ventilation tube into the myringotomy using forceps

### Questionnaire

Previously, we had tested individual software modules focusing on blade navigation [19], haptics [20] and tympanic membrane deformation and cutting [21]. Since this new simulator [22] refined each of these components, including the graphical representations of the ear and virtual tools, and included new features such as microscope handling, speculum positioning and tube insertion, the Myringotomy Surgery Simulation Scale (MS^3^) used in previous publications [20, 21] was modified to include these features. The questionnaire was divided into three sections (A, B, and C) with a total of 20 questions. Section A included 14 questions focusing on face validity as listed in Table 2. The appearance and realism of the surgical instruments; anatomy of the auricle, ear canal and eardrum; movement of surgical instruments; deformation and cutting of the eardrum; tube insertion and 3D microscopic view of the scene were assessed.

**Table 2:** Questions in Section A for face validity

No.	Question: Rate whether the following aspects of the simulator are realistic
1	Visual appearance of the auricle and ear canal
2	Visual appearance of the speculum
3	Movement of the speculum
4	Movement of the microscope/camera
5	Zoom of the microscope/camera
6	Visual appearance of the eardrum
7	Movement of the eardrum when physically contacted
8	Visual appearance of the myringotomy blade
9	Visual appearance and splay of the myringotomy
10	Visual appearance of the forceps
11	Movement and stability of the myringotomy blade and forceps
12	Visual representation of the tube
13	Movement of the tube within the myringotomy
14	Three-dimensional microscopic view of the scene based on light rendering, shadows, and 3D goggles

Section B included six questions focusing on content validity as listed in Table 3. These questions were used to determine training potential on specific surgical tasks.

**Table 3:** Questions in Section B for training potential

No	Question: Do you feel that the simulator would be useful in teaching Otolaryngology trainees the following skills
15	Speculum placement
16	Microscope positioning
17	Tool navigation
18	Ear canal and eardrum anatomy
19	Myringotomy creation
20	Tube insertion

In Sections A and B, study participants were asked to answer each question using a 7-point Likert scale, an equal appearing interval measurement. The scale had values of “1”—Strongly Disagree, 2—“Mostly Disagree”, 3—“Disagree”, 4—“Neither Agree/Disagree”, 5—“Agree”, 6—“Mostly Agree” and 7—“Strongly Agree”.

In Section C, a free-form comment area was provided for each participant to provide feedback to elaborate on previous questions and to address issues not covered in Sections A and B.

### Statistical analysis

The responses were initially divided by group (junior resident or practising Otolaryngologist), and the median, quartiles, minimum, and maximum response values were computed for each question. The sample size was maximized to include all eligible participants at a single academic institution. For each question, the Mann–Whitney *U*-test was used to test the significance of the differences in responses between the two groups. A frequency distribution histogram was plotted to investigate the number of favourable responses (score ≥ 5), neutral responses (score = 4), and negative responses (score ≤ 3) to each question. All data were computed and analysed using the SPSS statistical software (SPSS Inc, Chicago, IL). The significance was set at p ˂ .05 and the Holm-Bonferroni method was used to correct for multiple comparisons.

## Results

### Demographics

The first group was comprised of seven junior Otolaryngology residents in postgraduate years 1 to 3. They were all familiar with the operating microscope and the procedure, however they were in the active phase of learning with each resident having performed fewer than 20 myringotomy and tube insertions in training. The second group had five fellowship trained Otolaryngologists who routinely performed myringotomy and tube insertions in their practice. Each member of this group had performed at least 200 procedures since completing their fellowship.

### Comparison of groups

**Fig. 3:**
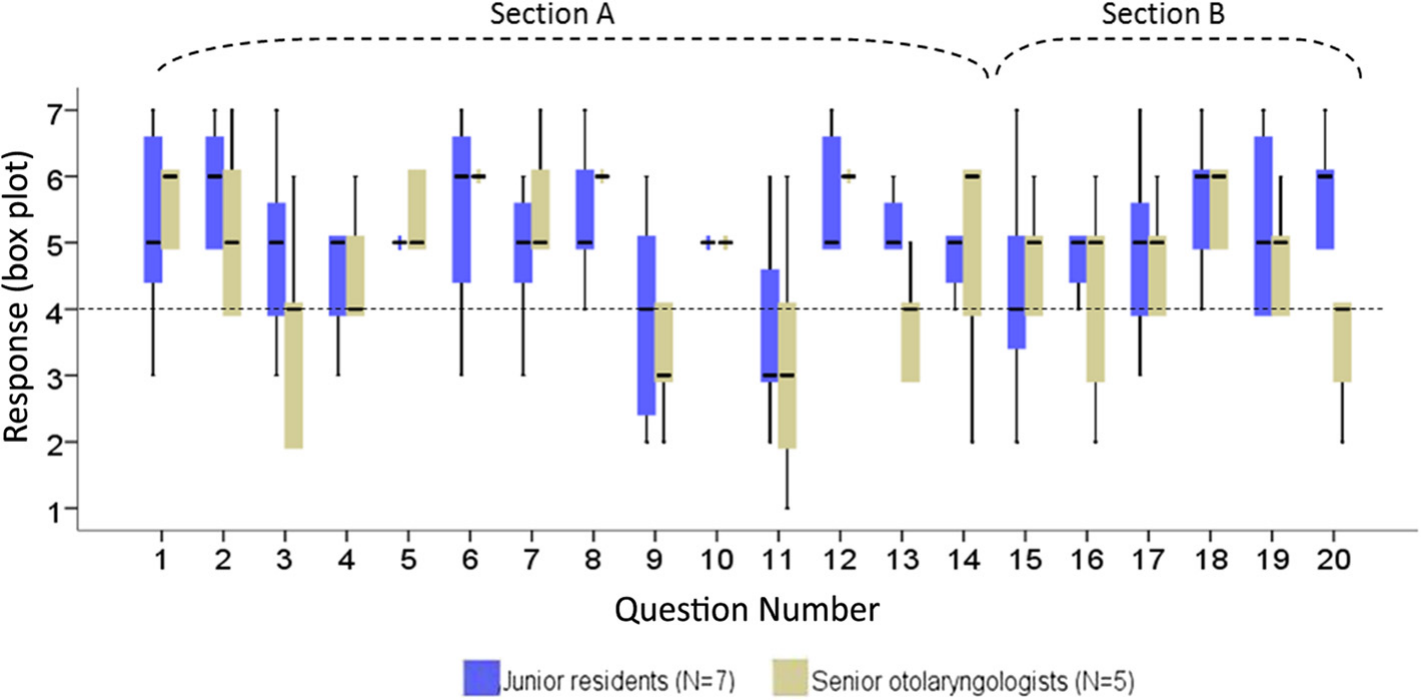
Box plot of the Likert item responses for the two groups of participants. Face validity was assessed in Questions 1–14, and content validity was assessed in Questions 15–20. A response of 4 is neutral, and higher values are more favourable than lower values

The mean response and confidence interval for each question in Section A (face validity) and Section B (content validity) are summarized in Fig. 3. Application of the Mann–Whitney *U*-test indicates no statistically significant differences between residents and senior Otolaryngologists once the Holm-Bonferroni correction was applied. However, the largest differences between the groups were seen in Question 13 (*U* = 5.5, *p* = 0.043) and Question 20 (*U* = 7, *p* = 0.097), which related to the movement of the tube within the myringotomy.

### Face and content validity

Given that mean responses were not different at the *p* = .05 level, the results for the two groups were pooled when analyzing face and content validity. The responses to the questionnaires were categorized as positive (score ≥ 5), neutral (score = 4) or negative (score ≤ 3).

### Face validity

**Fig. 4:**
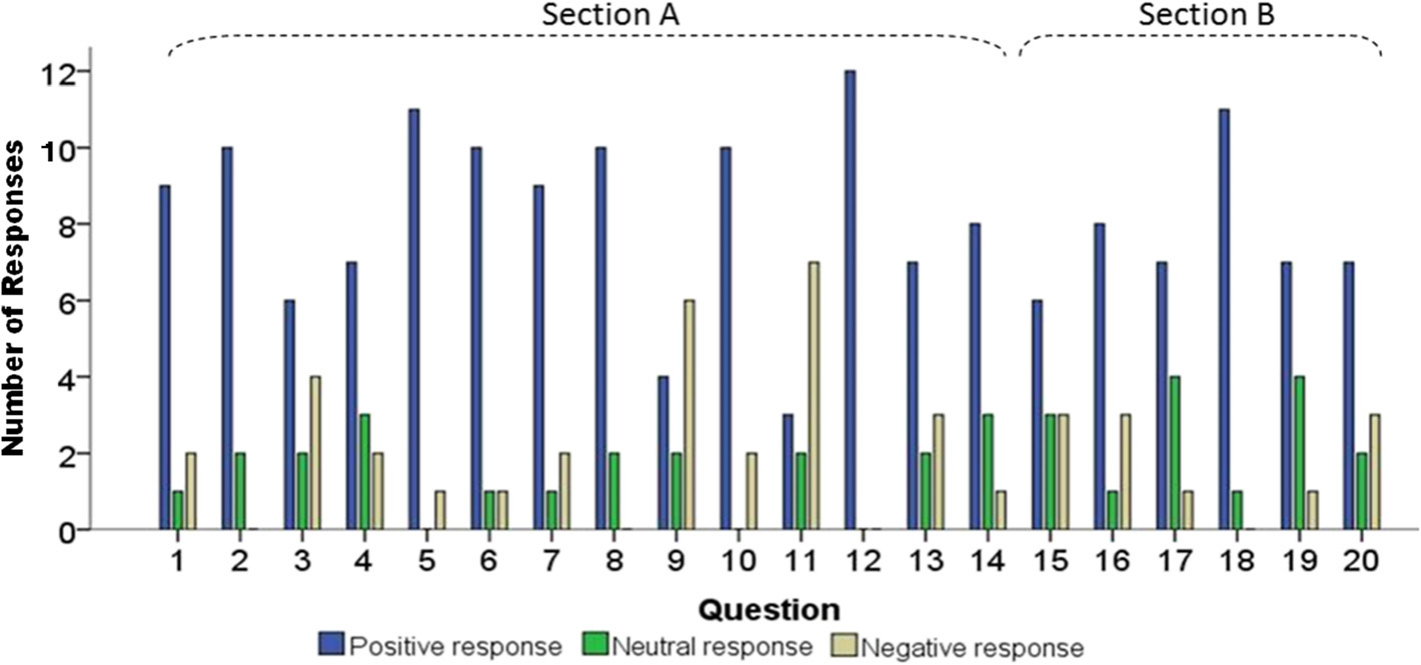
Total number of positive, neutral and negative responses to each question, pooling responses of junior residents and of senior Otolaryngologists. The blue bar indicates the number of positive responses (score ≥ 5), the green bar is the number of neutral responses (score = 4), and the beige bar indicates the number of negative responses (score ≤ 3)

The realism of the simulator was investigated through the 14 questions in Section A of the questionnaire. As can be seen in Fig. 4, the number of positive responses exceeds the number of neutral and negative responses except in the case of Questions 9 and 11. Question 9 focuses on the realism of the visual appearance and splay of the myringotomy, whereas Question 11 focuses on the realism of the movement and stability of the myringotomy blade and forceps. Overall, when the 14 questions over 12 participants (168 total responses) were considered, there were 116 (69.0 %) positive responses, 21 (12.5 %) neutral responses, and 31 (18.5 %) negative responses.

### Content validity

The training potential of the simulator was tested through 6 questions in Section B of the questionnaire. As shown in Fig. 4, the number of positive responses was greater than the number of negative responses for each question in this section. Among the total 72 responses (6 questions x 12 participants), 46 (63.9 %) were positive, 15 (20.8 %) were neutral, and 11 (15.3 %) were negative.

## Discussion

The MS3 scale used in this study had to be developed at our institution as no other validated measure was available to assess a virtual-reality myringotomy simulator. This questionnaire has not been externally validated by other centres, however content validity was assessed by a group of experts during the development of the questionnaire. In addition, previous publications [20, 21] did demonstrate reliability of the MS3 with a strong correlation across raters. The MS3 was also correlated against a visual analogue scale measuring the same construct, thus providing a measure of concurrent validity [21].

The lack of statistically significant differences in mean responses between residents and senior Otolaryngologists to Questions 1 to 20 suggests that even with limited exposure to the actual procedure of myringotomy with tube insertion, junior residents had similar assessments of the realism and utility of the simulator as those experienced in the OR.

The only differences between the groups approaching significance were in Questions 13 and 20, which pertained the movement of the tube within the myringotomy. Senior Otolaryngologists perceived the simulated tube movement to be less realistic than did residents. Similarly, Question 9 in the pooled responses dealt with the splay of the myringotomy, and this had a higher number of negative responses overall. From the written comments in Section C of the questionnaire, it appears that splaying (i.e., spreading) of the virtual eardrum when it is contacted by the virtual blade is realistic, and this was also the case in our previous report [21]; however, splaying is less realistic during tube insertion when the virtual tube contacts the eardrum and causes it to spread.

**Fig. 5:**
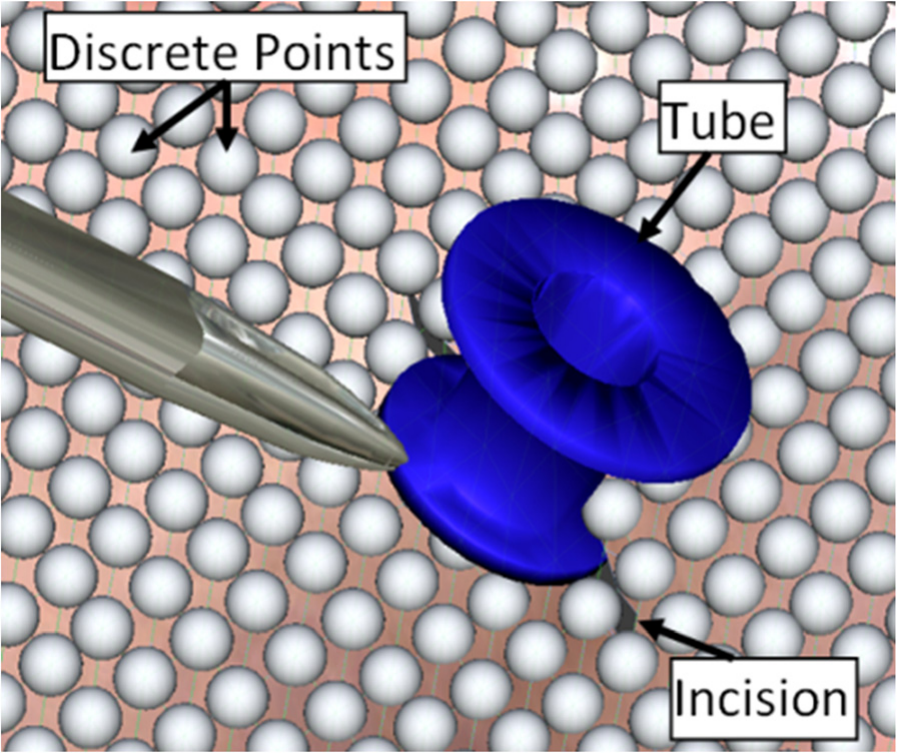
Representation of the virtual tympanic membrane by a collection of discrete points. The points define the geometry of the tympanic membrane and act as contact detectors with the virtual instruments (myringotomy blade, forceps, and ventilation tube)

This difference could be explained by a design decisions made during the development of the tube insertion module. First, although the tympanic membrane has real-time deformation, the physics of the interaction between the edges of the myringotomy and a ventilation tube is quite complex. In order to detect contact with the tube, the tympanic membrane is represented as a discrete collection of spatially distributed points as shown in Fig. 5. Collision detection is performed at each of these discrete contact points. When the spatial density of points is high (i.e. the points are close together) the location of contact can be calculated with more precision than when the spatial density is lower. Unfortunately, multi-point collision detection is computationally intensive, therefore the rendering speed decreases rapidly as the spatial density and precision is increased. The particular choice of density in the simulator was chosen to permit animations to occur at a realistic pace on an inexpensive personal computer, however this negatively affected the precision of the tympanic membrane splay in response to the tube.

Second, the physics of tympanic membrane ‘tearing’ with large forces and displacements during tube insertion are difficult to model in real-time. To overcome this, pre-programmed animations were used based on the length of incision, the trajectory of the tube, and the contact between the flange of the tube and the myringotomy. Although this significantly reduced computation time, Question 13 revealed that this lack of realism was noted by the experts and not the residents. This could be explained by the fact that senior surgeons would have had much more experience knowing how the ventilation tube *should* slide into the incision, therefore they were able to notice the subtle differences more than the junior trainees still learning the procedure. On average, Otolaryngologists’ rankings fell between “Disagree” to “Neither Agree/Disagree”, suggesting that slight improvements to the tube insertion simulation could make this aspect more acceptable.

Question 11 was the only other question with a higher proportion of negative responses, and this pertained to the movement and stability of the blade and forceps. Section C clarified this finding as concerns were raised about the limited range of motion of the haptic device and that the friction of the device affected the movements of the virtual blade and forceps. The haptic arm used in this study is a low-cost device that is suitable for design of a prototypical simulator. The device can easily be swapped for a higher fidelity device with greater range of motion and substantially reduced friction (e.g., Geomagic Phantom Premium device from Geomagic, Inc., Morrisville, NC), albeit at greater financial cost. Utilizing the higher fidelity device may result in acceptable range of motion and unnoticeable friction. A second concern with the device was the feel of the handle of the haptic arm when it was used to control the blade and forceps (Fig. 1). As the handle is thick, it feels unnatural compared to holding an actual surgical tool. We have implemented approaches described in the literature to replace the haptic arm handle with actual surgical tools to improve the feel and realism of the simulation [26]. The goal in this hybrid simulator would be have one haptic arm attached to a myringotomy blade or forceps, and have the second haptic arm attached to a real speculum to maximize realism.

Face and content validity are only initial steps in validation, and they do not ensure that a simulator will be useful in training residents [24, 25]. Future development on the Western myringotomy simulator will address concerns raised in this study. Refinement and optimization of the tube insertion and tympanic membrane splay may help to increase the realism of the simulator, but it is unclear if increased fidelity will actually result in additional skills transference [27]. In order to determine the construct validity of the simulator, automated metrics including time, length and direction of incision, collisions, magnification, etc. have been incorporated into the simulator. A separate study will examine if these metrics are capable of distinguishing experts from residents, and a skills transference study will be needed to determine if the simulator can result in better operating room performance. A multi-centred study will be considered at that time to maximize sample size and feedback from different centres.

The authors hope that by using standardized libraries while programming the simulator, and the ability of the simulator to run on low-cost hardware, will allow easy adoption by Otolaryngology training programs and allow other groups to make modifications as needed.

## Conclusion

The Western myringotomy simulator has a number of new features including microscope handling, speculum positioning and ventilation tube insertion. The simulator has good face and content validity, except with respect to splaying of the myringotomy during tube insertion and with respect to the haptic arm. These issues are currently being addressed with further refinements and adaptations. Automated metrics have been developed and they will be used to assess for construct validity of the simulator. Although the entire myringotomy and ventilation tube insertion can now be simulated, a skills transference study is needed to establish training efficacy and clinical impact.
